# Supplementary material for: Celldetective, an AI-enhanced image analysis tool for unraveling dynamic cell interactions
Source: eLife. 2026 Jul 8;14:RP105302. doi: 10.7554/eLife.105302 (PMC13345629; doi:10.7554/eLife.105302)
Supplement: Supplementary file 1. [file elife-105302-supp1.pdf]

Supplementary Tables

Table S1 Generalist deep learning segmentation models. This table lists the different generalist models (Cellpose or StarDist) which can be called natively in Celldetective. The sample images are cropped to (200 × 200) px and rescaled homogeneously to fit in the table.

name	modalities	# channels	dataset	sample image
CP_cyto3	cytoplasm nucleus	2	Cellpose [38] & user-submitted images	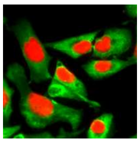
CP_livecell	cytoplasm (BF) black	2	LiveCell [40]	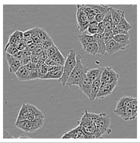
CP_tissuenet	cytoplasm nucleus	2	TissueNet [39]	/
CP_nuclei	nucleus black	2	DSB 2018 [78], Kaggle, ISBI 2009, MoNuSeg [79]	/
SD_versatile_fluo	nucleus	1	subset of DSB 2018 [78]	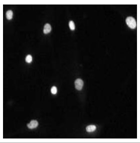
SD_versatile_he	H&E RGB	1	MonoNuSeg 2018 [79] TNBC 2018 [80]	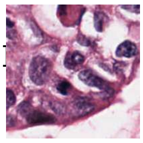

Table S2 MCF7 nuclei segmentation models in the presence of primary NK cells. Each model was trained on the same dataset of ADCC images, picking only the relevant channels.

Name	Channels	Type	Pretrained	Spatial calib. (μm)	sample image
mcf7_nuc_multimodal	Hoechst<br>Brightfield<br>CFSE<br>PI	StarDist	None	0.3112	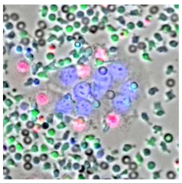
mcf7_nuc_stardist_transfer	Hoechst	StarDist	SD_versatile_fluo	0.3112	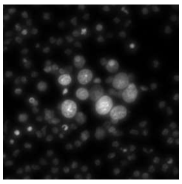

Table S3 Primary NK segmentation models. The models have been trained on a dataset of annotated primary NKs in ADCC images (*primary_NKs_w_MCF7*).

Name	Channels	Type	Pretrained	Spatial calib. (μm)	sample image
primNK_multimodal	Brightfield<br>CFSE<br>Hoechst	Cellpose	None	0.2178	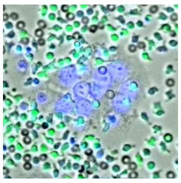
primNK_cfse	CFSE<br>None	Cellpose	CP_cyto2	0.2178	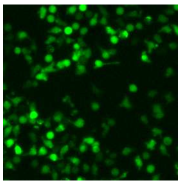
lymphocytes_ricm	RICM	Cellpose	None	0.2	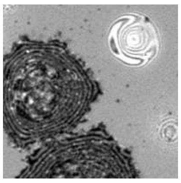

Table S4 Event detection models. We trained the following 1D DL models to classify and regress events of interest. The mean event response, centred at the event time, is shown for each channel in the pattern column.

Name	Signals	Task	Pattern
lysis_H_PI	Hoechst PI	Strong PI intake	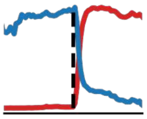
lysis_PI_area	PI area	Strong PI intake	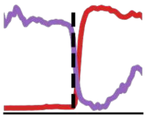
NucCond	area	Nucleus shrinking	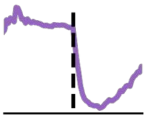
