# Supplementary material for: Celldetective, an AI-enhanced image analysis tool for unraveling dynamic cell interactions
Source: eLife. 2026 Jul 8;14:RP105302. doi: 10.7554/eLife.105302 (PMC13345629; doi:10.7554/eLife.105302)
Supplement: Peer review history

# Editor report: eLife Assessment

**DOI:** 10.7554/eLife.105302.3.sa0

**Authors:** Peter Koo

**Affiliations:** Cold Spring Harbor Laboratory United States

**Keywords:** Convincing, Important

CellDetective is an **important** software package for segmentation, tracking, and analysis of time-lapse microscopy datasets, specifically designed to be accessible to researchers without coding expertise. The authors provide **convincing** evidence of its capabilities through comprehensive validations and well-executed comparisons across immunological assays, and the latest version adds support for defining and visualizing multiple cell subsets. The current implementation remains limited to 2D widefield imaging, though the authors provide a sound rationale for this scope, and one interface issue (a fixed main-window size on some systems) still affects usability. Overall, this work will be of significant interest to the bioimaging community, especially those in immunology and cell biology, and has applicability extending well beyond immune profiling.

# Referee report: Reviewer #1 (Public review)

**DOI:** 10.7554/eLife.105302.3.sa1

Summary:

In this manuscript, Torro et al. presented CellDetective, an open-source software designed for a user-friendly execution of single cell segmentation, tracking and analysis of time-lapse microscopy data. The authors demonstrated the applications of the software by measuring NK cell spreading events acquired with reflection interference contrast microscopy (RICM), as well as detecting target cell death events and their interaction with neighboring NK cells in a multichannel widefield microscopy datasets.

Strengths:

The segmentation (StarDist, Cellpose) and tracking (bTrack) modules implemented were based on existing and published software packages, while the event detection, classification and analysis modules were added by the authors to enable an end-to-end time-lapse microscopy data processing and analysis pipeline, complete with graphical user interface (GUI) to minimize coding experience required from the user. The latest iteration of CellDetective also incorporates new features that enable multiple cell subsets to be examined and visualized. The documentation that accompanies CellDetective is also well written.

Weaknesses:

The current iteration of CellDetective is still limited to 2D 'widefield' analysis, although the authors have provided convincing justification for the current implementation for 2D + time analysis and clarified such limitations of the software in the manuscript. This reviewer maintains that support for 3D + time analysis in future iterations of CellDetective will substantially improve its applicability across broad disciplines, especially with emerging focus on 3D organoid studies.

Additionally, this reviewer has also encountered a key technical issue with the latest version of CellDetective (v1.5.2, installed on Windows 11 25H2) where the main CellDetective window is displayed in a fixed size that prevented the user from accessing the user interface/buttons that are essential for operating the software. As an example, in the very first demo (https://celldetective.readthedocs.io/en/latest/first-experiment.html), the fixed window size prevented this reviewer from accessing the "Submit" button in Step 2: Segment Cells (which is not visible as the fixed window size only displayed a certain portion of the GUI) of the workflow. This limitation made it near impossible to evaluate the useability and stability of the software. Fixing this issue by making the window size adjustable such that these buttons of the interface can be accessed by the user will be important to ensure the useability of the software.

This reviewer understands the difficulties and time involved in bug fixing, and hope that the experience could have been much smoother and the software behaves much more stably in order to maximize its useability.

# Author comment: Author response

**DOI:** 10.7554/eLife.105302.3.sa2

**Authors:** Rémy Torro, Beatriz Díaz-Bello, Dalia El Arawi, Ksenija Dervanova, Lorna Ammer, Florian Dupuy, Patrick Chames, Kheya Sengupta, Laurent Limozin

**Affiliations:** Aix-Marseille Univ, CNRS, INSERM, Turing Centre for Living systems Marseille France; Aix-Marseille Univ, CNRS, INSERM, Turing Centre for Living systems Marseille France; Aix-Marseille Univ, CNRS, INSERM, Turing Centre for Living systems Marseille France; Aix-Marseille Univ, CNRS, INSERM, Turing Centre for Living systems Marseille France; Aix-Marseille Univ, CNRS, INSERM, Turing Centre for Living systems Marseille France; Inserm Paris France; Aix-Marseille Univ, CNRS, INSERM, Turing Centre for Living systems Marseille France; Centre Interdisciplinaire de Nanoscience de Marseille Marseille France; Délégation Régionale Provence-Alpes-Côte d'Azur et Corse Marseille France

**Public Reviews:**

**Reviewer #1 (Public review):**

Summary:

In this manuscript, Torro et al. presented CellDetective, an open-source software designed for a user-friendly execution of single-cell segmentation, tracking, and analysis of time-lapse microscopy data. The authors demonstrated the applications of the software by measuring NK cell spreading events acquired with reflection interference contrast microscopy (RICM), as well as detecting target cell death events and their interaction with neighboring NK cells in a multichannel widefield microscopy dataset.

Strengths:

The segmentation (StarDist, Cellpose) and tracking (bTrack) modules implemented were based on existing and published software packages. The authors added the event detection, classification, and analysis modules to enable an end-to-end time-lapse microscopy data processing and analysis pipeline, complete with a graphical user interface (GUI). This minimizes the coding experience required from the user. The documentation that accompanies CellDetective is also adequate.

Weaknesses:

Given that the software was designed to improve user experience, such an approach also limits its scope and functionality and is currently capable of handling very specific types of experiments. Additionally, this reviewer has also encountered many technical difficulties (see documented bugs/crashes below) that have prevented an extensive exploration of all the functionality of CellDetective.

We thank the reviewer for recognizing the interest of the end-to-end pipeline design and the value of the graphical interface for non-coding users.

Scope and technical difficulties

We acknowledge the technical difficulties encountered during the review and sincerely apologize for the inconvenience. Since v1.3.9, we have invested substantial effort into stability, testing, and documentation. All reported bugs have been corrected and the software has been extensively tested (see points 4–7 below). Furthermore, in response to the concern about the software being limited to specific experiments, we note that Celldetective has since been successfully applied to other biological contexts beyond the immunological assays presented in the article, including microbiology (10.1128/mbio. 03342-25) and stem-cell-related studies (10.3390/jimaging11100371) (see also the positive remarks of Reviewer #2 regarding applicability). We also point the reviewer to the expanded documentation, which now includes modality-agnostic how-to guides.

Additionally, model transfer has been improved: retraining now freezes most layers by default, accelerating convergence and stabilizing fine-tuning for new datasets.

Specifics:

(1) The software can only handle 2D 'widefield' time-lapse imaging datasets. It should be noted that many studies that examine cell-cell interactions in vitro also used confocal microscopy and acquired the time-lapse images in 3D z-stacks to enable the reconstruction of entire cell volumes from multiple optical sections along the z-axis.

Given that almost all of the implemented segmentation (StarDist, Cellpose) and tracking (bTrack) packages already support the handling of 3D datasets, it is unclear why CellDetective was designed to only work with 2D datasets.

As noted above, extending the support for 3D images would allow the scope and utility of this software to be further extended for imaging studies acquired in z-stacks. As an example, the dense clustering of effector cells in Figure 4 had prevented accurate segmentation due to the 2D nature of the experimental dataset. More importantly, support for a 3D dataset could also allow for the tracking of fluorescent protein-based sub-cellular as well as membrane protein localization during cell-cell interactions.

Furthermore, it also widens the potential applicability for analyzing datasets from 3D organoid imaging and perhaps even intravital two-photon microscopy.

Scope and technical difficulties

We thank the reviewer for this suggestion and maintain our position that Celldetective is purposefully designed for high-throughput, high-temporal-resolution 2D imaging. We have now articulated this rationale more clearly in the revised manuscript (see Discussion lines 414-417).

Specifically, we emphasize that Celldetective's two core strengths — harnessing the statistical power of cell populations together with multiplexing biological conditions, and dynamic analysis of fast cellular events — both benefit from maximizing temporal resolution and field-of-view throughput. In our experience, Z-stack acquisition would reduce the achievable time resolution and throughput (in terms of captured events and parallel conditions) below acceptable levels for the minute-scale dynamics relevant to immunological assays.

That said, the modular architecture and the choice of 3D-compatible backends (StarDist, Cellpose, bTrack) leave the door open for community-driven 3D extensions in the future. We note in the revised manuscript that Celldetective is "specifically optimized for highthroughput, high-temporal-resolution imaging of quasi-2D systems" and that "by prioritising temporal sampling over Z-axis depth, Celldetective enables the capture of rapid biological dynamics that are often the focal point of interaction studies, where Z-stacking would otherwise limit throughput or resolution."

(2) The software in its current form only allows the broad demarcation of the cells examined into two populations: targets and effectors. This limits the number of cell populations that can be examined for their interactions. It might be more useful to just allow multiple user-defined populations instead of restricting the populations to target and effector cells only.

Extension to more than 2 custom populations

This has been fully implemented. Starting with version 1.4, Celldetective supports an arbitrary number of user-defined cell populations with user-chosen names. The restriction to "targets" and "effectors" has been removed. When creating a new experiment, users can now define any number of populations with custom labels (e.g., nk, rbc, macrophages, tumor_cells). The experiment configuration file stores this information in a generic [Populations] section. The control panel, segmentation, measurement, tracking, event detection, and neighbourhood modules all operate on these user-defined populations.

We illustrate this in the documentation with a figure showing a 3-population configuration (see the "How to create a new experiment" guide).

Neighbourhood analysis (interactions) currently supports pairwise interactions between any two populations (including same-population neighbourhoods), which covers the most common biologically relevant scenarios. We note that three-way (multipartite) interactions involve a substantially higher level of complexity and are, to our knowledge, not currently addressed by biologists in this context.

(3) Similarly, subsetting of each of the populations could be made more intuitive. Although it is possible to define subsets of cells using the "Custom classification" function under the "Measure" module with user-defined parameters, visualization of multiple groups remains unintuitive and it appears that only one custom classified group can be selected and visualized at any given time in the Signal Annotator under Measurement instead of allowing visualization of multiple (custom defined) groups of cells in different colors. It is also unclear how, if possible at all, to visualize a custom group of cells in the Signal Annotator under the Detect Events module.

Subsetting and visualization of multiple groups

The reviewer noted that defining cell subgroups through the Custom Classification was unintuitive, and that only one classified group could be visualized at a time.

We first clarify that Celldetective distinguishes between two visualization tools: the static measurement annotator (under Measure), which displays groups and characteristic groups on a per-frame basis, and the Event Annotator (under Detect Events), which displays event classes along temporal signal traces. The reviewer's request to visualize multiple customdefined groups in different colors falls under the measurement annotator.

We have addressed this concern with a multi-label characteristic group workflow: users perform successive threshold classifications to isolate individual phenotypes of interest (e.g., "spread", "dead", "high-intensity"), then merge these binary columns into a single characteristic group via the table view (Math → Merge states…). Each combination of states is automatically mapped to a distinct label and color. This merged column can then be explored in the measurement annotator, effectively displaying all subgroups simultaneously in different colors.

For more complex classification logic, the classification tool supports logical AND/OR operators for composing conditions, enabling flexible definition of subgroups without scripting.

The Event Annotator, by contrast, operates on a single event class at a time by design, as it is intended for reviewing and annotating individual event types along temporal signal traces; multi-group visualization is not applicable in this context.

Software issues:

(4) When initially tested on v1.3.9, the Segment module could not be initiated (with the error message AttributeError: 'WindowsPath' object has no attribute 'endswith' when attempting to run segmentation).

Update: this has been fixed in v1.3.9.post4 dated February 7th, 2025.

(5) Further testing was then performed by downgrading the software to v1.3.1. While testing the ADCC demo experiment (https://celldetective.readthedocs.io/en/latest/adcc-example.html), the workflow was stuck at attempts to initiate the Detect Events step:

AssertionError: No signal matches with the requirements of the model ['dead_nuclei_channel_mean', 'area']. Please pass the signals manually with the argument selected_signals or add measurements. Abort.

(Update: fixed in the latest v1.3.9.post4 version dated February 7th, 2025)

(6) Random bugs causing the software to crash. Example: switching characteristic to 'status_color' in the Signal Annotator under Measurement caused the software to crash (v1.3.9.post4):

TypeError: ufunc 'isnan' is not supported for the input types, and the inputs could not be safely coerced to any supported types according to the casting rule 'safe'

(7) Overall, when exploring the functionality of the software, there have been multiple instances of software crashes when clicking/switching around to show different parameters, etc.

This reviewer understands the difficulties and time involved in bug fixing and hopes that the experience could have been much smoother and that the software behaves much more stably in order to maximize its useability.

General stability — bug fixes and crash instances

We have made comprehensive improvements to software stability since the review period:

100+ bug fixes across v1.4.0–v1.5.0, systematically addressing crashes, edge cases, and error handling throughout the GUI.

Expanded automated test suite: the project now includes 43 test files (26 GUI-level tests + 17 unit test files) covering segmentation, tracking, measurements, event detection, filters, preprocessing, neighbourhoods, viewers, table operations, and more.

These tests run automatically via CI/CD on every commit.

Lazy imports for heavy dependencies (e.g., TensorFlow) to reduce startup time and potential import-order crashes.

Improved error handling: informative error messages instead of silent crashes; graceful fallbacks when optional dependencies are missing.

Usage and stability can be verified via GitHub traffic statistics and CI/CD action metrics.

**Reviewer #2 (Public review):**

Summary:

Immune assays enable the analysis of immune responses in vitro. These assays generate time series image data across several experimental conditions. The imaging parameters such as the imaging modality and the number of channels can vary across experiments. A challenge in the field is the lack of (open source) tools to process and analyze these data. R. Torro, et. al. developed an open source end-to-end pipeline for the analysis of image data from these immune assays. The pipeline is designed with a GUI and is suited for experimental biologists with no coding experience. The authors have incorporated several existing methods and tools for individual tasks such as for segmentation and cell tracking, and incorporated them with custom methods where necessary such as for tracking cell state transitions.

Strengths:

(1) The tool is extremely well-documented and easy to install.

(2) Applicable to a wide variety of imaging modalities and analysis.

(3) There are several different options for each step, such as segmentation using traditional methods or deep learning methods, and all the analysis steps are integrated in one place with a GUI. The no-coding requirement makes this a very powerful tool for biologists and has the potential to enable a wide variety of analyses.

We are grateful for the recognition of the tool's documentation quality, ease of installation, and versatility.

Weakness:

(1) It would be good to provide documentation on how to make the tool applicable for applications and analysis other than for immune profiling since most methods integrated here are applicable well beyond immune profiling. For example, a user might want to use the tool just for the segmentation of their IF microscopy-images.

Documentation for non-immune applications

We have undertaken a major documentation overhaul following the Diátaxis framework (Tutorials, How-to Guides, Explanations, Reference). The documentation now includes:

24 How-to guides covering individual tasks (segmentation, tracking, measurements, background correction, texture analysis, spot detection, channel alignment, survival analysis, interactions, event annotation, etc.), written in a modality-agnostic manner so that users from any application domain can follow them.

Concept pages explaining key abstractions (data organization, population-specific segmentation, single-cell events, survival, neighbourhoods) without assuming an immunology context.

Expanded tutorials, including the RICM spreading assay and the ADCC co-culture assay, which serve as worked examples that can be adapted to other biological systems.

The overview now presents Celldetective as "an open-source Python platform designed for biologists to study interacting cell populations in multimodal time-lapse microscopy", explicitly broadening the scope beyond immune profiling.

Additionally, the user-defined population naming (see Reviewer #1, point 2) naturally makes the tool more accessible to non-immunology users, as they are no longer constrained by "target/effector" terminology. The following articles from the literature refer to Celldetective in microbiology (10.1128/mbio.03342-25), for stem cells (10.3390/ jimaging11100371), or for CAR-T cells (10.1101/2025.06.24.661290v1, 10.1101/2025.07.25.666844v1), beyond the applications of this manuscript.

(2) They applied Celldetective to two immune assays. The authors present the results from these assays and use the results to validate their assay. However, they have not included data that demonstrates results obtained via this pipeline are comparable to results obtained with other pipelines and/or if these results are consistent with what is expected in the literature.

Comparison with other pipelines / literature validation

We emphasize that most of the presented data are original and do not have published equivalents, making direct pipeline-to-pipeline comparison impossible in many cases. We note that, to our knowledge, no existing open-source pipeline performs the complete endto-end analysis that Celldetective offers (from preprocessing through segmentation, tracking, event detection, neighbourhood analysis, to population-level survival curves), making a head-to-head software comparison impractical. Nevertheless, some recent publications have tested the software for various features (10.1128/mbio.03342-25, 10.1101/2025.07.25.666844v1), and results are in line with existing solutions when comparison is possible.

We reserve systematic comparison with traditional (non-microscopy-based) immunological assays for future dedicated studies, as we consider it out-of-scope for this software-focused manuscript.

Additional items for the revised manuscript

Manuscript changes (including private recommendations made by reviewers)

Modifications or additions in text appear in red:

Abstract: lines 15-17, 20-22, 24-26

Introduction: lines 71-72

Results: lines 91, 103, 127-137, 170-171, 196-201, 239-242, 250-252, 255-257, 261-264,

266-269, 292-295, 303, 319-321

Figure captions fig.1, fig. 2, fig. 3, fig. 5

Discussion: 372-377, 384-387, 406-407, 414-417, 418

Materials and Methods: lines 462-464, 542-546, 673-677, 684-685, 733-734

Figure S10

References have been updated.

Article statistics (as of 30 Apr 2026)

2799 views

162 downloads

7 citations

**Recommendations for the authors:**

**Reviewer #1 (Recommendations for the authors):**

Minor points:

(1) For the study involving LAMP1 measurement, a representative image of LAMP1 antibody staining should be included.

We added a new supplementary figure Fig S10 with reference to it on line 304

(2) In Figure 5B, can the authors comment on the ostensibly higher effector velocity under HER2+ target conditions? Is this caused by variation within assay, and whether they have been confirmed in independent wells/experiments with the same conditions?

Thanks to the reviewer for this remark; We have added a comment on lines 320-322

As we don’ t have systematic replicates for this effect, we tentatively attribute it to a variation in the target cell coverage.

(3) It is not clear why in the Signal Annotator under Measurement, the movie playback is performed with a user-draggable slider but in the Signal Annotator under Detect Events, the movie plays with only a Play/Stop button with no options to modify the playback speed or to advance the movie frame-byframe.

This has been addressed in Celldetective v1.5.0. The Signal Annotator for event detection now provides frame-by-frame navigation buttons, an autoplay mode for natural playback of dynamics, and on-the-fly animation speed control, matching the functionality available in the Measurement viewer.

**Reviewer #2 (Recommendations for the authors):**

(1) Main text

One major comment throughout the manuscript is that the experimental setup sections (ie lines 143149; 221-226 and interspersed in the section 'From a single time point to a dynamic readout of effector-target interactions') are hard to follow. It is clear that the tool can achieve what is described, but it is hard to follow why the experiments were set up that way and it took digging through methods and figure captions to understand what the setup was in terms of antibodies (what are the specificity, why are they chosen, what are they proxies for). Each section has some of that information but not jointly. It would help to have a high-level description of the experimental aim and then how this has been achieved in the setup with details on the antibodies, including targets and what their role would be. This would help with for instance understanding what the purpose of the PI stain as a measure introduced in line 245 is, or how the antibodies in experiment one relate to the ones in experiment 2, etc. The hardest part to parse was the section on effector-target interactions, specifically how the simulation is set up and why. The clarity of the manuscript could really benefit from a reworking of these paragraphs.

The mentioned paragraphs have been rewritten following the reviewer’s suggestions.

Lines 127-137: The RICM assay intro has been substantially rewritten in red, providing high-level experimental aim, bsAb function, surface preparation, and RICM rationale — all in a single coherent block.

Lines 196-201: The ADCC assay intro is rewritten in red with clear description of bsAb purpose, cell types, HER2 variation, PI monitoring, and fluorescent labels.

In the same spirit, we also added a biological context to introduce the last section of results on lines 261-264.

It is not clear what implications the statement in line 267 has for the user.

A comment was added on lines 239-242

In line 191, it is stated that the position-based approach showed a spike that was not observed in the mask-based approach. It is not clear what the spike means, is it an artifact or a real phenomenon discovered by the position-based approach; this is important as the t_spread definition would differ depending on which segmentation is used

A comment was added on line 167. It does not impact the definition of t_spread since the peak is observed during the spreading phase.

In the co-culture assay, StarDist approach is used to segment the MCF7 cell line while Cellpose is to segment the NK cells. Please provide a rationale for selecting these differing approaches for segmentation.

A justification was added on lines 265-267

The impact of cell density was looked at for 32 micrometers, however, it is not clear why this cut-off was chosen.

A justification was added on lines 250-252

(2) Methods

Lines 743/744: what type of manual adjustments? If important for usable, should be described in detail.

Details have been added on lines 674-678

If specifying what software was used for plots, then also mention which ones are used for exceptions.

Details are provided in a new dedicated paragraph, lines 735-739

(3) Discussion

Conclusion in line 404 - direct protective effect, or just sampling effect?any data for either, or too strong a conclusion otherwise.

We have added a short discussion on this topic, lines 372-377.

Preliminary analysis of ADCC rates stratified by local target density and number of effector neighbours suggests that both factors contribute (unpublished data), and Celldetective's neighbourhood analysis module provides the tools to perform such stratified survival studies.

I don't understand the implications in line 412, maybe just the wording choice. Prior studies in T cells could not resolve, but would now be feasible with celldetective? Or for T cells this is still not possible due to other experimental constraints?

Thanks for this remark; indeed it could not be resolved yet for T cells, to our knowledge, but would be facilitated by celldetective.

A comment was added on lines 385-388

(4) Figures

(a) Font sizes in all figures are generally too small.

Fonts in all figures have been enlarged.

(b) Figure 2

F, G, H: clarify caption.

F: single cells grey traces, average colored line?

G: what's the confidence/error interval?

H: State the statistic and meaning of the qualitative assessment.

DONE

(c)Figure 3:

F/G: choice of 3.5 as neighbouring cell is not motivated; mode would have been at 4 and choosing a non-integer for cell counts seems strange from a biological perspective.

A comment has been added in the caption.

E/G: what is the error/confidence interval?

DONE

(d) Figure 5:

A: error bars?

ADDED

(5) Minor typos/word choices

(a) Typo in line 59 - double the.

OK

(b) Typo in line 214 - upper case U in middle of sentence.

OK

(c) Typo/word choice in 516/17 - cells were split? Kept instead of keep.

OK

Typo 706; missing space between time and using.

OK

All corrected
